# Life Cycle Assessment of Fused Filament Fabrication Using Recycled Plastic and Carbon Fiber Composites

**DOI:** 10.3390/polym18050660

**Published:** 2026-03-08

**Authors:** Kautilya Patel, Rutva Sheth, Shashikant Joshi, Dhaval Shah

**Affiliations:** Mechanical Engineering Department, Institute of Technology, Nirma University, S-G Highway, Ahmedabad 382481, India; 21ftphde54@nirmauni.ac.in (K.P.); 21bme129@nirmauni.ac.in (R.S.); s.j.joshi@nirmauni.ac.in (S.J.)

**Keywords:** life cycle analysis, 3D printing, sustainability, Open LCA

## Abstract

This study presents a comprehensive investigation into the application of Life Cycle Assessment (LCA) for advanced manufacturing and recycling processes, with a focus on achieving sustainability goals. The environmental and economic impacts of additive manufacturing (AM) and innovative recycling strategies for materials like carbon fiber-reinforced plastics (CFRPs) and 3D printing polymers are analyzed. Experimental efforts detail the preparation of recycled plastic–carbon fiber composite filaments suitable for Fused Filament Fabrication (FFF). The composite exhibits enhanced mechanical, thermal, and flame-resistant properties through optimal blending of plastic waste and carbon fibers. Sustainability assessments using Open LCA 2.2.0 and SolidWorks 2022 demonstrate significant environmental benefits aligned with circular economy principles. The analysis highlights that the weight reduction results in lifetime fuel savings combined with end-of-life credits of −1.32 kg CO_2_-eq for composite core versus +0.10 kg CO_2_-eq for plastic parts. The recycled composite achieves a net global warming potential of −12.55 kg CO_2_-eq, compared to +2.44 kg CO_2_-eq for plastic components. The study emphasizes challenges such as recyclability, material degradation, and regional applicability of global LCA models, while proposing pathways for future advancements.

## 1. Introduction

The rise of industrialization and rapid population growth over the last few decades have fueled a demand for large-scale production, which has come at a considerable environmental cost. Among the many forms of waste generated, plastic waste has emerged as one of the most severe and persistent threats to ecosystems and human health. The scale of the problem is staggering: according to the Central Pollution Control Board (CPCB), India generated 8.782 million metric tons of hazardous waste in 2019–2020, of which the state of Gujarat alone contributed nearly 28%, or 2.485 million metric tons [1]. Out of this, only 1.225 million metric tons were recycled, indicating a pressing gap in effective waste management systems. On a global scale, approximately 8 million metric tons of plastic enter the world’s oceans every year, devastating marine ecosystems and contributing to the formation of microplastics [2]. These tiny particles, resulting from the breakdown of larger plastic debris, are now found in water bodies, agricultural soil, the air, and even in human food chains, raising significant concerns about their long-term effects on health and biodiversity. This growing crisis highlights the urgent need for innovative and sustainable strategies in manufacturing and waste utilization.

In response to these challenges, sustainability has become a central driver of innovation across industries. The transition from traditional production methods to environmentally conscious technologies is not only a necessity but also an opportunity for reshaping manufacturing practices. One of the most widely accepted frameworks for guiding this transition is Life Cycle Assessment (LCA), which provides a standardized methodology to evaluate the environmental, economic, and social impacts of products across their entire life cycle—from raw material extraction to end-of-life disposal. LCA enables researchers, policymakers, and industries to identify critical intervention points where resource consumption can be reduced, waste can be minimized, and recycling can be optimized [3]. Moreover, it provides a foundation for decision-making aligned with global sustainability goals, such as the reduction of carbon emissions, resource efficiency, and the promotion of a circular economy.

In the field of manufacturing, several advanced technologies have been examined through the lens of LCA for their sustainability potential. For instance, Laser Beam Machining (LBM) has shown promise in specialized production contexts. A study by Hankammer et al. [4] reported that only 4% of the overall cost of LBM processes is attributed to energy consumption, making it comparatively efficient. Its suitability for low-volume, high-variance production allows for material precision and reduced wastage. However, its industrial adoption at scale remains limited by technical complexity and initial investment costs, necessitating further optimization for broader sustainability impacts.

Injection molding is a mature and low-defect manufacturing process widely used for high-volume polymer composite production. It provides excellent material consolidation and dimensional stability [5]. However, it requires expensive tooling and is less suitable for decentralized or low-volume recycling applications. Additive manufacturing (AM), although more energy intensive, offers tooling-free production, geometric flexibility, and compatibility with recycled materials. Sam-Daliri et al. [6] demonstrated significant recovery of tensile and flexural properties in injection-molded parts prepared from recycled AM waste, which is used to support the claim that injection molding can mitigate inherent defects introduced during layer-wise fabrication.

AM, popularly known as 3D printing, has attracted significant attention for its potential contributions to sustainable production [7,8,9,10]. Unlike subtractive methods, which involve cutting away material and often result in significant waste, AM builds components layer by layer, thereby reducing material usage. It also enables decentralized and flexible supply chains, which can reduce transportation emissions and support localized production. A study by Ford et al. [11] emphasized that AM processes tend to be energy-intensive, especially when using certain polymeric and metallic feedstocks. While it provides benefits like mass customization, labor reduction, and design flexibility, these gains are often offset by inefficiencies in energy consumption. Comprehensive LCA studies are therefore essential to balance these trade-offs and guide the sustainable development of AM technologies.

Another critical factor in sustainable manufacturing is material selection. The environmental footprint of a product is heavily influenced by the raw materials used and their recyclability. Saade et al. [12] compared different 3D printing feedstocks and found that UV resin-based printing exhibited lower environmental impacts compared to Acrylonitrile Butadiene Styrene (ABS), while biodegradable polymers such as Polylactic Acid (PLA) and recyclable options like Polyethylene Terephthalate Glycol (PETG) occupied an intermediate position. Furthermore, process choice has been shown to affect overall efficiency: Stereolithography (SLA) was found to be more efficient than Fused Deposition Modeling (FDM) in both material usage and energy consumption. These findings underscore the importance of not only selecting sustainable materials but also optimizing the processes used to manufacture them [13].

Carbon Fiber-Reinforced Polymers (CFRPs) represent another class of materials gaining attention for their balance of strength, lightweight properties, and potential sustainability benefits when recycled [14]. Widely used in the aerospace, defense, and automotive industries, CFRPs contribute to fuel efficiency and performance improvements due to their high strength-to-weight ratio. However, their end-of-life management has traditionally posed challenges. Recent studies, however, have provided promising insights. For example, Stelzer et al. [15] demonstrated that replacing carbon fibers with recycled fibers significantly reduced environmental impact, particularly when advanced recycling techniques such as irradiation and plasma treatment were employed. LCA conducted in Singapore further highlighted that thermochemical conversion methods, such as pyrolysis, were more effective than conventional waste-to-energy techniques in transforming plastic waste into valuable fuels [14,16]. These approaches illustrate how technological innovation in recycling can unlock sustainability benefits from materials previously considered difficult to reuse.

The literature on fiber-reinforced FFF shows that short carbon fibers significantly improve tensile strength and stiffness while maintaining processability using standard extrusion systems. Continuous-fiber systems provide higher strength but require specialized hardware and complex alignment control. Therefore, short-fiber systems provide a practical balance between mechanical performance and manufacturing feasibility.

The recycling and reusability of biodegradable plastics also continue to be areas of exploration. PLA, one of the most commonly used biodegradable polymers in 3D printing, faces challenges due to viscosity loss after multiple recycling cycles, limiting its utility to two recycling iterations. Saroj et al. [17] proposed that blending recycled PLA with PLA could enhance recyclability and extend its life cycle. Karimi et al. [18] investigated the potential of using polybutylene adipate-co-terephthalate for direct pellet printing, eliminating the need for filament conversion with optimal printing parameters. Despite these advancements, limitations remain, particularly regarding scalability and cost-effectiveness. Furthermore, the impact of these methods varies across regions due to differences in ecological systems and resource availability. For instance, Testa et al. [19] and Hankammer et al. [4] emphasized the importance of localized LCA models that account for biodiversity, water availability, and land use, in addition to global indicators such as greenhouse gas emissions.

Taken together, the reviewed literature emphasizes that sustainable manufacturing requires a multidimensional approach. Optimized material selection, efficient recycling technologies, and localized LCA models must converge to create systems that are not only environmentally friendly but also economically viable and socially beneficial. It is within this broader framework that the present study situates itself. This paper explores the development of a novel composite material composed of 50 wt.% LDPE, 25 wt.% HDPE, 5 wt.% MAPE and 20 wt.% carbon fiber, with potential applications in additive manufacturing and beyond. By integrating waste plastic into high-performance composites, this research aims to address two pressing issues simultaneously: reducing plastic pollution and creating sustainable, functional materials for modern manufacturing. In doing so, it seeks to contribute to circular economy goals and provide a pathway toward scalable solutions that balance environmental protection with industrial progress.

## 2. Materials and Methods

Plastic waste is one of the most pressing environmental challenges due to its non-biodegradable nature, improper disposal, and contribution to microplastic pollution in soil, water, and even the food chain. Traditional waste management strategies have struggled to cope with the scale of the problem, making it imperative to explore innovative recycling pathways. In this context, additive manufacturing (AM) offers a sustainable alternative to conventional methods. By building components layer by layer, AM reduces material wastage, enables lightweight designs, and supports decentralized production, thereby lowering resource consumption and emissions.

**Figure 1:**
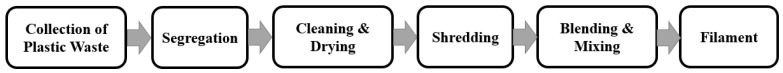
Proposed methodology to prepare a novel material.

To address sustainability goals, a novel composite material was developed using 50 wt.% LDPE, 25 wt.% HDPE, 5 wt.% MAPE procured from Arran chemical company Ltd. and Zoltek made 20 wt.% carbon fiber. Recycled plastic contributes to waste valorization, while carbon fiber enhances mechanical properties due to its high strength-to-weight ratio and durability. This eco-friendly blend not only diverts plastic from landfills but also provides a robust, lightweight, and sustainable feedstock for AM applications. The proposed methodology to prepare this material is shown in Figure 1.

### 2.1. Collection of Plastic Waste

**Figure 2:**
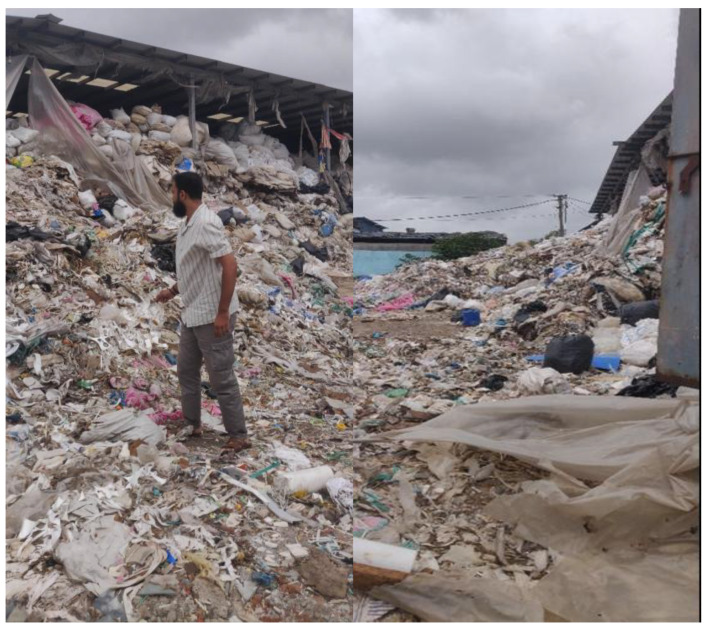
Collection of plastic waste from various sources.

Plastic waste was collected from various sources such as households, packaging waste, industrial leftovers, and discarded containers as shown in Figure 2. The goal here was to gather mostly polyethylene plastics (LDPE and HDPE) since they melt well, bond with carbon fiber using additives, and are commonly found in everyday waste. It is important to avoid mixing plastics like PVC or PET at this stage, as they require different processing conditions and can ruin the final product. Industrial-scale separation methods such as density-based separation and rejection of PET and PVC streams were considered to ensure polymer purity.

### 2.2. Segregation of Plastic Types

**Figure 3:**
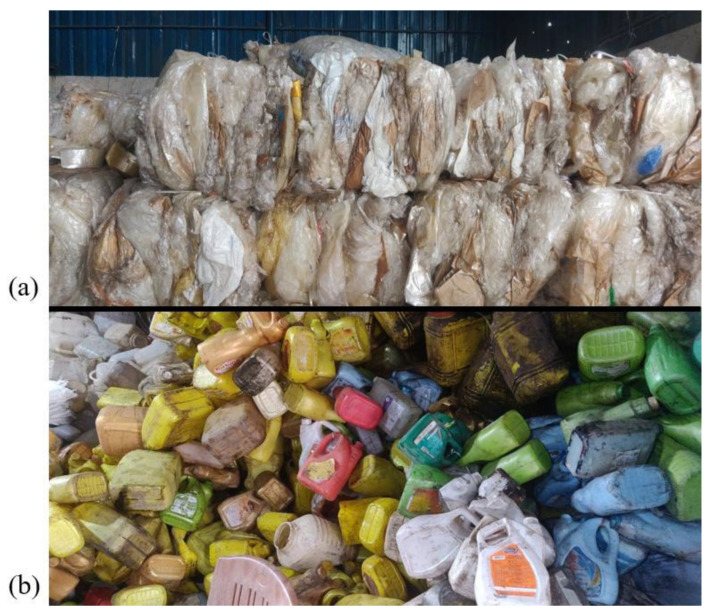
Segregation of plastic types (**a**) LDPE and (**b**) HDPE.

Once collected, the plastic waste was sorted manually based on type as shown in Figure 3. LDPE (used in plastic bags and films) and HDPE (used in containers and bottles) were separated. Segregation ensures uniform melting and avoids processing issues later. Visual inspection and recycling codes (like “2” for HDPE and “4” for LDPE) helped us identify the correct plastic. Non-plastic materials like labels, rubber, and metal parts were removed to avoid contamination.

### 2.3. Cleaning and Drying of Plastic Waste

Plastics were thoroughly cleaned using warm water at 70 °C mixed with sodium carbonate (soda ash) and a mild detergent after sorting. This solution breaks down oily residues, dirt, and any organic matter stuck to the plastic. The plastic was stirred for 30 min in this solution and then rinsed with clean water to remove leftover chemicals. The cleaned plastic was dried completely using sunlight or mechanical dryers. Plastics were cleaned and dried at 60–80 °C for 4–6 h to eliminate moisture prior to extrusion. Moisture must be eliminated completely to avoid bubbles or degradation during melting.

### 2.4. Shredding of Collected Plastic Waste

**Figure 4:**
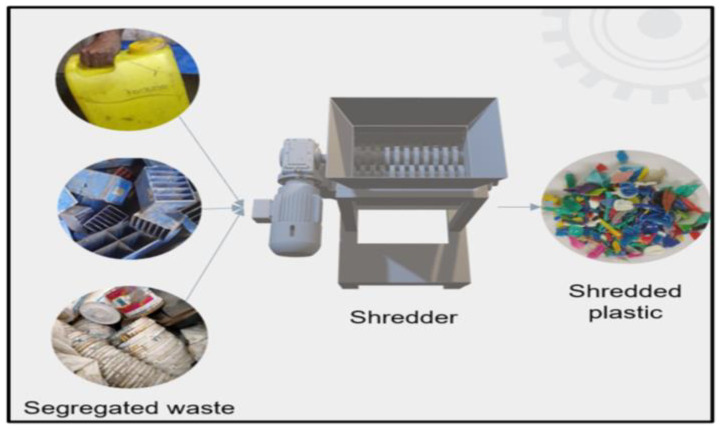
Shredding of HDPE and LDPE using mechanical shredder.

The dried plastic was shredded into small flakes, about 5–10 mm in size, using a mechanical shredder available at Siloxane Aggrandize Innovative Industries as shown in Figure 4. Smaller and uniform pieces melt evenly and help in proper mixing with additives and carbon fibers. Shredding also increases the surface area, allowing better bonding with the carbon fibers. This shredding process reduces the particle size of plastic waste, thereby increasing the surface-to-volume ratio and enhancing heat transfer during processing. This reduction in particle size facilitates more uniform and rapid melting in the extruder, resulting in decreased melting time and improved processing efficiency.

### 2.5. Blending and Mixing of Chopped Carbon Fibers

**Figure 5:**
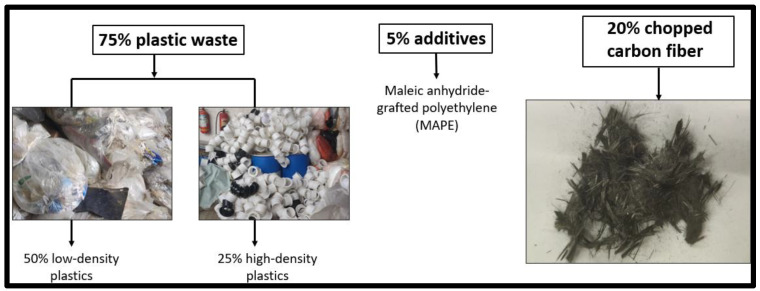
Blended plastic waste with chopped carbon fiber.

The preparation of the composite material began with shredded plastic waste, which was carefully proportioned to achieve the desired mechanical and processing characteristics. The mixture consisted of 50% Low-Density Polyethylene (LDPE), selected for its flexibility and smooth melt flow, combined with 25% High-Density Polyethylene (HDPE) to provide strength and stiffness. To enhance processing and bonding characteristics within the composite, 5% Maleic anhydride-grafted polyethylene (MAPE) was incorporated as an additive. In recycled polymer systems, excessive compatibilizer content may also lead to phase softening and reduced stiffness. Therefore, 5% MAPE represents a practical and optimized concentration, offering effective interfacial bonding while maintaining stable extrusion, printability, and mechanical reliability. Finally, 20% chopped carbon fibers (3 mm length) were added to reinforce the plastic base, ensuring improved strength and reduced weight of the final material, as shown in Figure 5. All components were measured by weight and thoroughly blended using a high-speed mixer to ensure uniform dispersion of fibers and additives throughout the polymer matrix. Achieving a homogeneous mixture was essential to guarantee consistent material properties. In cases where industrial mixing equipment was unavailable, manual mixing in smaller batches was employed, ensuring careful and prolonged agitation to achieve an even distribution of constituents.

### 2.6. Extrusion Process for Filament Preparation

**Figure 6:**
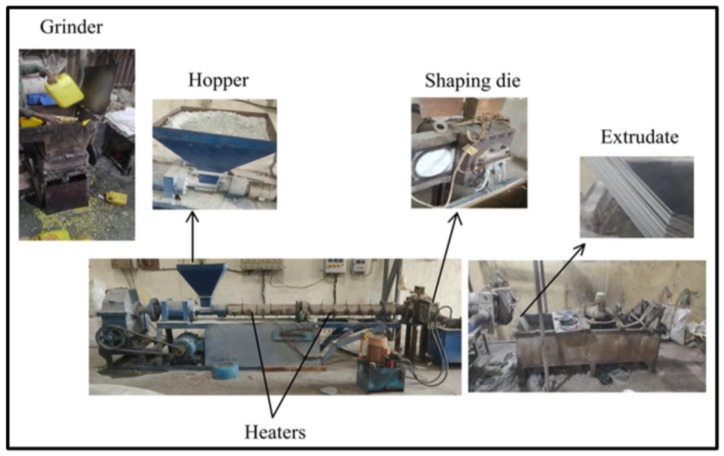
Filament preparation process using extrusion process.

The mix was inspected for dryness and uniformity after blending. It was then stored in airtight containers or directly loaded into the extrusion machine, as shown in Figure 6. Moisture and uneven mixing at this stage can cause filament breakage, air pockets, or weak bonding in the final product. Proper preparation here ensures smooth filament formation or part molding during extrusion.

### 2.7. Fabricated Filament for 3D Printing

**Figure 7:**
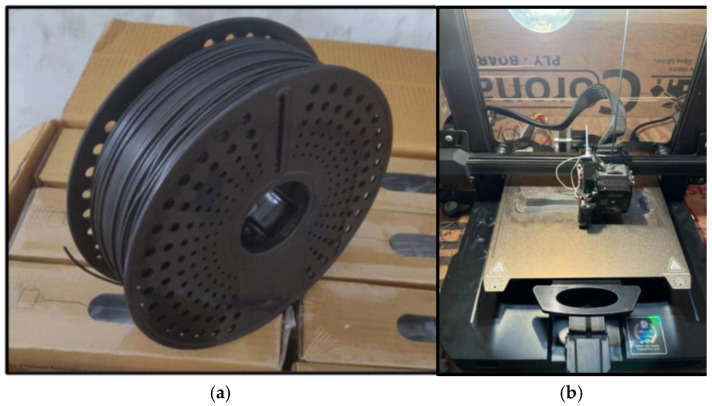
(**a**) Fabricated extruded filament used for 3D printing; (**b**) FDM printing composite filament.

The filament extruder type and other operating and printing condition parameters are depicted in Table 1. This method transforms common plastic waste into a high-performance composite material suitable for use in 3D printing, lightweight structural parts, or prototyping, as shown in Figure 7. The carbon fiber reinforcement, along with the additive and mixed plastic base, creates a material that is not only sustainable but also mechanically superior to recycled plastic alone.

Filament was produced using a single-screw extruder at 170–190 °C. A 1.75 mm filament diameter was maintained. Printing was performed using FFF with a 0.4 mm nozzle, 200–220 °C nozzle temperature, 50 mm/s speed, 0.1–0.3 mm layer height, 100% linear infill, and flat orientation. These filaments are designed for compatibility with FDM technology, enabling the fabrication of test specimens with very high dimensional precision and accuracy.

## 3. Results

### 3.1. Material Life Cycle Assessment (LCA)

This section presents a cradle-to-cradle Life Cycle Assessment (LCA) for a composite material composed of 50 wt.% LDPE, 25 wt.% HDPE, 5 wt.% MAPE and 20 wt.% carbon fiber, manufactured through segregation, cleaning, shredding, and blending. The purpose of conducting this assessment is to quantify the environmental impact of producing the composite and compare its performance with conventional materials such as virgin plastics and Carbon Fiber-Reinforced Polymers (CFRPs). By considering the material across its entire life cycle, the study highlights the potential of recycled composites to promote sustainability, reduce waste, and support circular economy principles.

The goal of this assessment is to evaluate the environmental burdens associated with producing 1 kg of the composite material. The focus is on identifying emissions, resource use, and other environmental consequences arising from its production and disposal. Using 1 kg as the reference point allows for direct and consistent comparison with other commonly used materials such as plastics and CFRPs. Ultimately, the goal is not only to quantify impacts but also to identify hotspots in the process where improvements can be implemented to make the material more sustainable.

The scope of the study adopts a cradle-to-grave approach, which accounts for every stage of the product’s life cycle. This includes the collection and processing of waste plastic, involving sorting, cleaning, shredding, and blending; the manufacturing stage, where the composite is prepared and formed into usable material; and the end-of-life stage, which considers multiple treatment pathways such as recycling, incineration, or landfill disposal. By including all stages of the life cycle, the scope ensures that no significant environmental impacts are overlooked and that the analysis provides a holistic understanding of the material’s sustainability profile.

The functional unit of the study is defined as 1 kg of composite material. This unit serves as the basis for comparison with other materials and ensures that results are expressed in a consistent and meaningful way. The system boundaries extend from raw material acquisition to end-of-life treatment, covering all inputs and outputs across the life cycle. This includes sourcing post-consumer plastic waste and industrial carbon fiber, the energy consumed during sorting, cleaning, shredding, and blending, as well as emissions arising from these processes. Additionally, the system incorporates the environmental consequences of disposal methods, whether through recycling loops, incineration, or landfill. By clearly defining the system boundaries and functional unit, the assessment ensures methodological consistency and reliability in evaluating the composite’s overall environmental footprint.

### 3.2. Life Cycle Inventory (LCI)

The Life Cycle Inventory (LCI) stage compiles the material and energy flows necessary for the production of the functional unit [15,20]. For the composite under study, this includes 0.8 kg of recycled plastic and 0.2 kg of carbon fiber. The recycled fraction is obtained from post-consumer plastic waste streams, while carbon fiber is sourced from industrial processes. Electricity used in cleaning, shredding, blending, and mixing is also included, along with auxiliary additives that enhance bonding and processing. This inventory forms the basis for impact assessment, enabling a quantitative evaluation of the composite’s environmental performance relative to other materials.

Next, energy consumption is analyzed during each step of processing, as given in Table 2. This is based on small-to-medium scale semi-automated operations and reflects practical power usage in developing countries like India. The total energy consumption is 0.70 kWh per kg of composite material.

### 3.3. Life Cycle Impact Assessment (LCIA)

The environmental impact—specifically in terms of global warming potential (GWP) and water footprint—is evaluated after collecting inventory data as shown in Table 3. Under the cut-off approach, recycled materials entering the system boundary are assigned zero upstream environmental burden associated with their original production. GWP measures the total greenhouse gas emissions (in kg CO_2_-equivalent) associated with each stage of production and disposal [21,22,23]. The emissions primarily arise from carbon fiber production and electricity usage, while recycled plastic is credited with zero emissions due to its diverted waste origin. Table 3 shows that 91% of the total emissions come from the carbon fiber, highlighting it as the main impact contributor. The water consumption is also a vital sustainability indicator, especially in water-scarce regions beyond GWP as given in Table 4. The composite has a lower water footprint compared to materials due to the high percentage of recycled plastic and relatively dry processing methods.

### 3.4. End-of-Life Analysis (LCIA)

End-of-life (EoL) treatment plays a significant role in reducing the overall environmental burden of any material. For this composite, the product is assumed to undergo three treatment routes at the end of its life: recycling (50%), incineration (40%), and landfill disposal (10%), as shown in Table 5. These treatment paths are based on realistic recycling and disposal patterns in semi-urban Indian settings. Recycling provides a negative emission credit, as it offsets the need for material in future production cycles. Incineration contributes slightly to emissions, while landfill impact is relatively negligible [24,25].

### 3.5. Final Cradle-to-Grave Global Warming Potential

Combining both production emissions and end-of-life credits gives us the final net GWP for the composite material. This is the most critical indicator for sustainability comparison. The final GWP summary is given in Table 6. This result demonstrates that the composite has a total carbon footprint of 5.05 kg CO_2_-eq per kg, which is significantly lower than traditional carbon fiber composites.

### 3.6. Evaluation of Findings for LCA

The LCA clearly identifies carbon fiber as the dominant contributor to emissions, accounting for more than 90% of total GWP. Recycled plastic, on the other hand, brings minimal or even beneficial impact, especially when considering its diversion from landfills. The Indian electricity grid, although relatively carbon-intensive, contributes a smaller share due to low overall energy consumption (0.70 kWh/kg). However, improvements such as switching to solar-powered processing could further reduce emissions. The total production GWP was 6.37 kg CO_2_-eq/kg. The end-of-life credit was −1.32 kg CO_2_-eq, resulting in a net GWP of 5.05 kg CO_2_-eq/kg.

End-of-life management plays a major role in offsetting emissions. A 50% recycling rate offers a 21% reduction in GWP, reinforcing the importance of designing recyclable composite products. Compared to conventional CFRP materials, which have a carbon footprint of around 25–30 kg CO_2_-eq per kg, this recycled composite provides an emission saving of up to 80%, making it a much more environmentally responsible option for structural, consumer, or industrial applications.

## 4. Case Study: Comparing Armrest Made of Polypropylene (PP) vs. Recycled Composite

**Figure 8:**
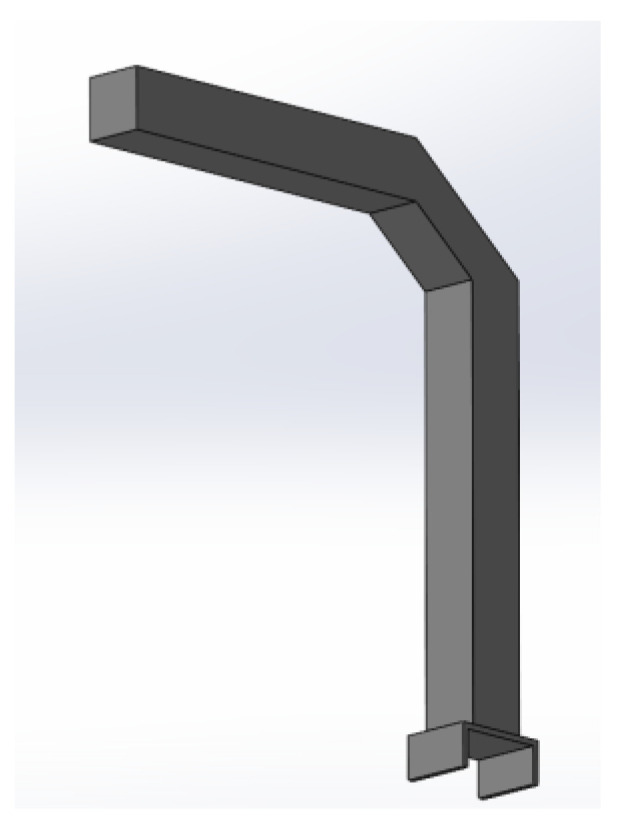
3D CAD model of armrest.

This study evaluates the environmental and mechanical implications of replacing a conventional PP armrest core with a recycled composite alternative in automotive applications. The 3D CAD model of the armrest is prepared in solid modeling software as shown in Figure 8. The recycled composite core demonstrates a substantial improvement in mechanical performance compared to the polypropylene core. Each specimen was printed three times to ensure consistency. Tensile testing is carried out according to ASTM D638 Type I specifications [26], and flexural testing is performed following ASTM D790 [27] procedures. With the tensile strength being nearly three times higher and the flexural modulus more than double, it provides superior stiffness and load-bearing capability. At the same time, the composite is ~16.7% lighter, contributing to significant weight savings without compromising impact resistance. These properties make the material highly attractive for lightweight automotive applications, where both structural efficiency and sustainability are critical design requirements. The detailed comparison of various parameters for the PP core and recycled composite core is given in Table 7.

### 4.1. Goal and Scope

The goal of this assessment is to quantify the environmental benefits achieved when substituting a conventional polypropylene (PP) armrest core with a lighter recycled composite alternative. The evaluation specifically focuses on emissions generated over the expected vehicle lifetime of approximately 200,000 km, taking into account how reduced component mass translates into fuel savings and lower greenhouse gas emissions.

The scope of the study follows a cradle-to-grave perspective, which encompasses the entire life cycle of the armrest core—from raw material production and manufacturing to its use-phase within the vehicle and eventual end-of-life (EoL) treatment. Since both the PP and recycled composite cores perform the same structural and functional role, they are considered functionally equivalent, ensuring that the comparison is fair and unbiased.

The functional unit for this analysis is defined as one armrest core with an equivalent mass of approximately 1 kg. The system description includes raw-material sourcing (petrochemical-based polypropylene for the baseline and recycled plastic with carbon fiber reinforcement for the composite); molding processes; the operational phase of the vehicle, where weight reduction leads to fuel efficiency gains; and finally, the EoL pathways, including recycling, incineration, or landfill. This comprehensive approach ensures that all significant life cycle stages are captured for accurate environmental assessment.

### 4.2. Life Cycle Inventory for Armrest

The LCI aggregates inputs and outputs linked to material weight, emissions, and benefits, as given in Table 8. Based on PP cradle-to-gate 5300 L per 1000 kg, equivalent to ~5 L/kg, the production GWP, fuel saving factor, and other parameters are taken from various sources like eeer.org, americanchemistry.com, cartonplast.com, and plasticmakers.org.

### 4.3. Life Cycle Impact Assessment for Armrest

**Figure 9:**
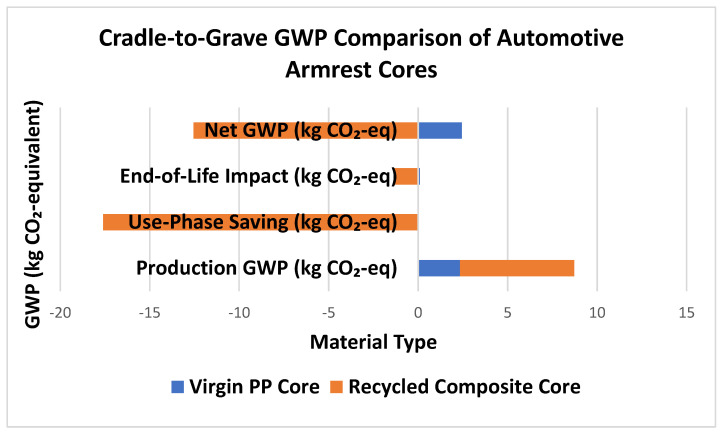
Comparison of automotive armrest cores.

The LCA quantifies greenhouse gas impacts across the product lifespan. The production of the recycled composite results in 172% higher emissions compared to polypropylene (6.37 kg CO_2_-eq vs. 2.34 kg CO_2_-eq), primarily due to the inclusion of carbon fiber and energy-intensive mixing processes, as given in Table 9. However, the weight reduction of 0.2 kg per part enables substantial fuel savings over the vehicle’s 200,000 km lifetime. This translates to a use-phase benefit of approximately 17.6 kg CO_2_-eq avoided emissions. Additionally, the composite’s higher recyclability (50%) and reduced landfill contribution give it a more favorable end-of-life profile, offsetting another 1.32 kg CO_2_-eq. Despite its higher initial production emissions, the recycled composite achieves a net-negative GWP of −12.55 kg CO_2_-eq, making it a significantly more sustainable alternative to PP in long-term automotive applications. The comparison of automotive armrest cores is shown in Figure 9.

### 4.4. End-of-Life Analysis for Armrest

The end-of-life (EoL) stage plays a crucial role in determining the overall environmental footprint of both the polypropylene (PP) core and the recycled composite core [28,29]. For the PP core, an assumed distribution of 30% recycling, 60% incineration, and 10% landfill results in a net positive impact of approximately +0.10 kg CO_2_-eq. In contrast, the recycled composite achieves more favorable outcomes due to higher recyclability rates. With 50% of the composite being recycled, 40% incinerated, and 10% landfilled, the result is a net negative impact of −1.32 kg CO_2_-eq. This demonstrates that enhanced recyclability and the ability to recover material value at the EoL stage can significantly improve the sustainability of composite-based products compared to conventional polymers.

### 4.5. Evaluation of Findings for Armrest

Both materials were assessed across mechanical, operational, and environmental performance metrics to provide a holistic comparison. Mechanical properties used in the armrest comparison are application-level benchmarks derived from validated composite datasets to support LCA-driven substitution analysis. Mechanically, the recycled composite shows substantial improvements: its tensile strength (68 MPa) is nearly three times higher than that of the PP core (25 MPa), while its flexural modulus (2860 MPa) is more than double the PP counterpart (1360 MPa). Despite these gains, the impact strength of both materials remains comparable at 9 kJ/m^2^, ensuring that the composite does not sacrifice toughness for stiffness. Additionally, the composite achieves a weight reduction of 0.2 kg (1.0 kg vs. 1.2 kg), which directly contributes to improved efficiency in lightweight design applications.

From an environmental standpoint, the results are equally compelling. While the production of the composite core incurs a higher initial global warming potential (GWP) of +6.37 kg CO_2_-eq compared to +2.34 kg CO_2_-eq for PP, this disadvantage is offset by the composite’s performance during the use-phase. The 0.2 kg weight reduction translates to fuel savings equivalent to –17.6 kg CO_2_-eq over a vehicle lifetime of 200,000 km, resulting in significant environmental benefits. Combined with end-of-life credits of −1.32 kg CO_2_-eq, the composite achieves a net GWP of −12.55 kg CO_2_-eq per core, whereas the PP core ends with a net GWP of +2.44 kg CO_2_-eq. This highlights the ability of the recycled composite to deliver a net-negative carbon footprint, thereby actively contributing to emission reductions.

Water footprint analysis shows that the composite consumes more water during production (~15 L/kg) compared to PP (~5 L/kg). However, given the scale of emissions savings, the relative increase in water use remains modest and does not outweigh the significant carbon reduction benefits. The tensile strength improved from 25 MPa to 68 MPa, and the flexural modulus from 1360 MPa to 2860 MPa. The net life cycle GWP was −12.55 kg CO_2_-eq compared to +2.44 kg CO_2_-eq for PP. Overall, the composite core not only demonstrates superior mechanical performance and weight efficiency but also provides a markedly improved environmental profile, making it a promising alternative to conventional PP components in automotive applications [25,30,31].

## 5. Discussion

The goal of this study was to evaluate a recycled composite material, made from 50 wt.% LDPE, 25 wt.% HDPE, 5 wt.% MAPE and 20 wt.% carbon fiber, as a sustainable substitute for conventional polypropylene (PP) in automotive armrest cores. The focus was on determining whether such a material could deliver superior mechanical performance while simultaneously reducing environmental impacts across its life cycle.

The results indicate that the recycled composite significantly outperforms PP in terms of strength and stiffness. With a tensile strength of 68 MPa and flexural modulus of 2860 MPa, the composite is almost three times stronger and more than twice as stiff as PP, while maintaining comparable impact resistance. Furthermore, it achieves a 0.2 kg weight reduction (1.0 kg vs. 1.2 kg), translating to a ~16.7% mass saving. For automotive applications, this improvement in structural efficiency directly supports lightweight design, which is essential for fuel efficiency and performance.

From an environmental perspective, the analysis highlights an important trade-off. Although the composite exhibits a higher production footprint (+6.37 kg CO_2_-eq) compared to PP (+2.34 kg CO_2_-eq), the benefits become clear during the use-phase. Weight reduction results in lifetime fuel savings equivalent to −17.6 kg CO_2_-eq over 200,000 km. When combined with end-of-life credits (−1.32 kg CO_2_-eq versus +0.10 kg CO_2_-eq for PP), the recycled composite achieves a net GWP of −12.55 kg CO_2_-eq, compared to +2.44 kg CO_2_-eq for PP. This demonstrates that the composite not only offsets its higher production impacts but ultimately delivers a net-negative carbon footprint.

Overall, the discussion confirms that integrating recycled plastics with carbon fiber reinforcement can provide both mechanical advantages and significant environmental gains. The material’s balance of lightweight efficiency, durability, and recyclability makes it a strong candidate for sustainable automotive applications.

## 6. Conclusions

This study demonstrates that the recycled plastic–carbon fiber composite core is a technically robust and environmentally superior alternative to polypropylene (PP) in automotive interior applications such as armrests. The incorporation of 50 wt.% LDPE, 25 wt.% HDPE, 5 wt.% MAPE and 20 wt.% chopped carbon fiber not only addresses the pressing issue of plastic waste management but also results in a high-performance composite that delivers significant engineering and ecological benefits. The enhanced mechanical properties, including a tensile strength nearly three times that of PP and a flexural modulus more than double, enable weight savings of ~16.7%, which in turn translate into meaningful reductions in fuel consumption and use-phase emissions.

Although the recycled composite exhibits a higher initial production footprint compared to PP, the overall Life Cycle Assessment highlights its superiority. Over a 200,000 km vehicle lifetime, the composite achieves a net-negative carbon footprint (−12.55 kg CO_2_-eq), primarily due to fuel efficiency and higher recyclability at end-of-life. This finding demonstrates that the material not only offsets its production burden but also contributes positively to climate impact mitigation. Furthermore, its alignment with circular economy principles—valorizing plastic waste and reducing reliance on virgin petrochemicals—strengthens its relevance in the context of global sustainability goals.

Beyond environmental advantages, the study underscores an important paradigm shift: sustainability and performance need not be mutually exclusive in material design. The composite validates that recycled materials, when intelligently engineered, can surpass traditional counterparts in strength, durability, and efficiency. These results suggest strong potential for wider adoption of such composites in automotive and other lightweight engineering applications.

In conclusion, the recycled plastic–carbon fiber composite offers a compelling pathway toward greener, stronger, and lighter material solutions. Future research could focus on scaling production, exploring fatigue and thermal behavior, and expanding applications across the transportation and construction industries to maximize its sustainability impact.

## Figures and Tables

**Table 1:** Filament and printing parameters.

Process Stage	Parameter	Specification/Value
Filament Extrusion	Screw configuration	Single screw
	Extrusion temperature	170–190 °C
	Feedstock form	Shredded LDPE–HDPE blend with chopped carbon fiber
	Carbon fiber content	20 wt.%
	Average fiber length	~3 mm
	Filament diameter	1.75 mm
3D Printing (FFF)	Printer type	Creality make material extrusion (FFF) 3D printer ender-5
	Nozzle diameter	0.4 mm
	Nozzle temperature	200–220 °C
	Printing speed	50 mm/s
	Layer height	0.1–0.3 mm
	Infill pattern	Linear
	Infill density	100%
	Print orientation	Flat orientation

**Table 2:** Processing energy consumption during each stage.

Process	Energy Use (kWh/kg)
Segregation	0.10
Cleaning	0.15
Shredding	0.20
Blending	0.25
Total	0.70

**Table 3:** Emission factor and total GWP for material.

Source	Unit	Emission Factor (kg CO_2_-eq/Unit)	Total GWP (kg CO_2_-eq)
Recycled Plastic	0.8 kg	0.0 (credited)	0.00
Carbon Fiber	0.2 kg	29.0	5.80
Electricity (India Grid Mix)	0.7 kWh	0.82	0.574
Total GWP	—	—	6.37

**Table 4:** Water footprint.

Material	Water Use (L/kg)
Plastic	~20
CFRP	~100
Composite Material	~15

**Table 5:** End-of-life emission breakdown.

Treatment	Share (%)	Emission Factor (kg CO_2_-eq)	GWP Contribution
Recycling	50	−3.0	−1.50
Incineration	40	+0.4	+0.16
Landfill	10	+0.2	+0.02
Total	—	—	–1.32

**Table 6:** Final GWP summary.

Stage	Value (kg CO_2_-eq)
Material Production	+6.37
End-of-Life Credit	−1.32
Net Total	5.05

**Table 7:** Comparison of various parameters for PP core and recycled composite core.

Parameters	PP Core	Recycled Composite Core
Material Type	Polypropylene	50% LDPE + 25% HDPE + 5% MAPE + 20% Carbon Fiber
Weight	1.2 kg	1.0 kg
Manufacturing Process	Injection Molding	Compression/Extrusion Molding
Expected Lifespan	~10 years	~10 years
Tensile Strength	~25 MPa	~68 MPa
Flexural Modulus	~1360 MPa	~2860 MPa
Izod Impact Strength	9 kJ/m^2^	9 kJ/m^2^
Material Origin	Petrochemical-Based	Recycled Plastic + Carbon Fiber
Mass Reduction Benefit	—	~16.7% Lighter (0.2 kg Saving)
Structural Efficiency	Moderate	High (Due to Fiber Reinforcement)

**Table 8:** Life Cycle Inventory parameters for armrest made using PP core and recycled composite core.

Parameters	PP Core	Recycled Composite Core
Material Weight	1.2 kg	1.0 kg
Production GWP	+2.34 kg CO_2_-eq ^1^	+6.37 kg CO_2_-eq
Use-Phase Saving	0	–17.6 kg CO_2_-eq ^2^
End-of-Life GWP	+0.10 kg CO_2_-eq ^3^	–1.32 kg CO_2_-eq
Water Footprint	~5 L (PP production)	~15 L (composite ‡)
Net Total GWP	+2.44 kg CO_2_-eq	–12.55 kg CO_2_-eq

‡ Composite water use includes minimal washing + processing. ^1^ PP GWP: 1.95 kg CO_2_-eq/kg × 1.2 kg (jpoll.ut.ac.ir) ^2^ Fuel-saving: 200,000 km × −0.38 L/100 km/100 kg × 1 kg × 2.31 kg CO_2_/L (jpoll.ut.ac.ir) ^3^ PP EoL: Incineration + landfill, estimated +0.10 kg CO_2_-eq; composite uses previous end-of-life breakdown.

**Table 9:** Environmental Life Cycle Impact Assessment of the armrest system.

Material	Production	Use Saving	EoL Impact	Net GWP
PP Core	+2.34	0	+0.10	+2.44 kg CO_2_-eq
Composite Core	+6.37	−17.6	−1.32	−12.55 kg CO_2_-eq

## Data Availability

The raw data supporting the conclusions of this article will be made available by the authors on request.

## Abbreviations

The following abbreviations are used in this manuscript:

LCA	Life Cycle Assessment
LCI	Life Cycle Inventory
LCIA	Life Cycle Impact Assessment
GWP	Global Warming Potential
CO_2_-eq	Carbon dioxide equivalent (climate impact unit)
kWh	Kilowatt-hour (unit of energy)
CFRP	Carbon Fiber-Reinforced Polymer
ABS	Acrylonitrile Butadiene Styrene (plastic type)
EoL	End-of-Life
kg	Kilogram
L	Liters (used in water footprint)
km	Kilometer
